# Hepatoprotective Activity of Herbal Composition SAL, a Standardize Blend Comprised of* Schisandra chinensis*,* Artemisia capillaris*, and* Aloe barbadensis*

**DOI:** 10.1155/2016/3530971

**Published:** 2016-03-15

**Authors:** Mesfin Yimam, Ping Jiao, Breanna Moore, Mei Hong, Sabrina Cleveland, Min Chu, Qi Jia, Young-Chul Lee, Hyun-Jin Kim, Jeong-Bum Nam, Mi-Ran Kim, Eu-Jin Hyun, Gayoung Jung, Seon Gil Do

**Affiliations:** ^1^Unigen, Inc., 3005 1st Avenue, Seattle, WA 98121, USA; ^2^Unigen, Inc., No. 450-86, Maebong-Ro, Dongnam-Gu, Cheonan-Si, Chungnam 330-863, Republic of Korea; ^3^Univera Inc., No. 78, Achasan-ro, Sungdong-gu, Seoul 04775, Republic of Korea

## Abstract

Some botanicals have been reported to possess antioxidative activities acting as scavengers of free radicals rendering their usage in herbal medicine. Here we describe the potential use of “SAL,” a standardized blend comprised of three extracts from* Schisandra chinensis*,* Artemisia capillaris*, and* Aloe barbadensis*, in mitigating chemically induced acute liver toxicities. Acetaminophen and carbon tetrachloride induced acute liver toxicity models in mice were utilized. Hepatic functional tests from serum collected at T24 and hepatic glutathione and superoxide dismutases from liver homogenates were evaluated. Histopathology analysis and merit of blending 3 standardized extracts were also confirmed. Statistically significant and dose-correlated inhibitions in serum ALT ranging from 52.5% (*p* = 0.004) to 34.6% (*p* = 0.05) in the APAP and 46.3% (*p* < 0.001) to 29.9% (*p* = 0.02) in the CCl_4_ models were observed for SAL administered at doses of 400–250 mg/kg. Moreover, SAL resulted in up to 60.6% and 80.2% reductions in serums AST and bile acid, respectively. The composition replenished depleted hepatic glutathione in association with an increase of hepatic superoxide dismutase. Unexpected synergistic protection from liver damage was also observed. Therefore, the composition SAL could be potentially utilized as an effective hepatic-detoxification agent for the protection from liver damage.

## 1. Introduction

Liver diseases as a result of habitual repeated alcohol consumption, exposure to some xenobiotics, and/or drug interactions are some of the major causes of morbidity and mortality where variable symptoms manifested ranging from asymptomatic elevation of the liver enzyme to sudden hepatic failure. Liver is susceptible to alcohol-induced injury as both alcohol and its primary metabolite acetaldehyde produce reactive oxygen species (ROS) and hydroxyl radicals (OH), altering hepatic antioxidant defense system [1]. While damage of oxidative stress affects the whole body as a system, the impact becomes more detrimental when it involves vital organs such as the liver where primary detoxification takes place to remove and metabolize harmful toxins such as alcohol. The most frequently observed pathological conditions of the liver such as fatty liver, hepatitis, fibrosis, and cirrhosis are common findings in alcohol-linked liver disorders as a result of recurrent exposure of alcohol. These outcomes in conjunction with cellular lipids, proteins, and DNA oxidation have been demonstrated in multiple experimental animal models [2]. Among these models, the acetaminophen and carbon tetrachloride induced hepatotoxicity models have been most frequently used in assessing hepatoprotective activity of nutraceuticals.

Various xenobiotics are known to cause hepatotoxicity, among which acetaminophen (n-acetyl-p-aminophenol or APAP) and carbon tetrachloride (CCl_4_) are generally utilized to develop an animal model that mimics the human type of liver toxicity with similar mechanisms of actions. Acetaminophen (n-acetyl-p-aminophenol, APAP, also known as Tylenol, Paracetamol) is a very safe and effective analgesic and antipyretic drug at therapeutic dosage. However, APAP overdose can cause severe liver toxicity characterized by depletion of GSH, protein adduct formation [3, 4], generation of highly active free radicals, mitochondrial damage, and nuclear DNA fragmentation [5] that leads to cell death and hence necrosis. While some species like rat are relatively resistant to APAP toxicity, the mouse is the preferred model as several studies have demonstrated dose dependent response to either oral or intraperitoneal APAP challenge [6, 7]. Similarly, CCl_4_, a halogenated alkane with restricted usage as industrial chemical/solvent, is a well-known hepatotoxin that is widely used to induce acute toxic liver injury in a large range of laboratory animals. CCl_4_ toxicity is initiated by cytochrome P450s (CYP) primarily of 2E1 [8], to yield reactive metabolic products trichloromethyl free radicals (CCl_3_^−^), which can initiate lipid peroxidation and ultimately results in the overproduction of reactive oxygen species (ROS) and hepatocyte injuries [9, 10]. These radicals can also react with oxygen to form the trichloromethylperoxy radical CCl_3_OO^−^, a highly reactive species that could initiate the chain reaction of lipid peroxidation leading to cell death. Therefore, it could be inferred that, regardless of the chemical agents used to induce the hepatotoxicity, both the acetaminophen and carbon tetrachloride models share the critical step in oxidative stress induced by reactive oxygen species generated by excess intermediate metabolites leading to protein oxidation, lipid peroxidation, and DNA damage.

Historically, some botanicals containing phenolic compounds have been reported to be associated with antioxidative actions in biological systems, acting as scavengers of free radicals rendering their usage in herbal medicine. We hypothesized that combining such plant materials with historical efficacy and safety data would give a beneficial boost in their indication for overall liver health. In the screening process, we selected and tested 38 plant extracts (plants list not shown) collected through legacy mining leading to the discovery of a composition designated as SAL which comprised* Schisandra chinensis*,* Artemisia capillaris*, and* Aloe vera*.

*Schisandra chinensis*, also known as Wuweizi and Wurenchum, is traditionally used for conditions of lung and kidney insufficiency. It is also indicated in cases of chronic cough and dyspnea, diarrhea, night sweats, wasting disorders, irritability, palpitations, and insomnia, as well as a general tonic for treating fatigue associated with illness [11]. In modern pharmacotherapy, mounting experimental and clinical evidences suggest the hepatoprotective nature of* Schisandra* extracts preventing carbon tetrachloride induced hepatotoxicity and glutathione depletion and stimulating the activity of glutathione reductase [12–14]. The major active principles of* Schisandra* are lignans called schisandrins, which have energizing properties by increasing the activity of some enzymes which participate in the oxidative phosphorylation process and also increased superoxide dismutase and catalase activities in rat liver cytosol and were able to inhibit gossypol-induced superoxide anion generation in rat liver microsomes [15]. The hepatoprotective effect of* Schisandra* fruit extracts has been reported in Chinese literature for the treatment of patients with hepatitis, in a clinically controlled trial resulting in 68% (72/107) and 44% (36/72) improvement in serum ALT levels within 4 weeks and 8 weeks [16].

*Artemisia capillaris*, with the common name “yinchen” or “yinchenhao” in Chinese depending on the different collection season, also known as “yinjin” in Korean, is one of the commonly used TCM included in various ancient Chinese dispensatories. The earliest record of* Artemisia capillaris* was recorded in* Shen Nong Ben Cao Jing* (*The Classic of Herbal Medicine*)—a Chinese book of agriculture and medicinal plants—for treating jaundice, removing the dampness, and use as a diuretic. Both aqueous extracts and ethanol extracts have been reported with hepatoprotective efficacy in both* in vitro* assays and* in vivo* animal studies [17, 18]. Catechins, coumarins, flavonoids, organic acids, water soluble polysaccharides, and polypeptides have been reported as active components responsible for the liver protective activities of* Artemisia capillaris* [18].

*Aloe vera* N-931 is a composition containing a unique combination of 1–4% aloesin and 96–99% 200 : 1* Aloe vera* inner leaf gel powder with not less than 8% polysaccharides blended via a conventional method (Aloecorp, USA). Chromones isolated from various* Aloe* species have been reported to have diverse biological activity. A C-glycosyl chromone isolated from* Aloe barbadensis* demonstrates anti-inflammatory activity [19] and antioxidant activity similar to that of alpha-tocopherol based on a rat brain homogenates model [20]. Aloesin is a C-glycosylated 5-methylchromone with a potent antioxidation activity [21].

Here we implemented the most frequently used animal model with practical clinical implications such as acetaminophen and confirmed findings with the classic carbon tetrachloride induced hepatotoxicity model to assess the effect of the composition SAL in protecting liver from such damage. In addition, the merit of combining* Schisandra chinensis*,* Artemisia capillaris*, and* Aloe barbadensis* was also evaluated.

## 2. Materials and Methods

### 2.1. The Composition

SAL is a novel synergistic composition containing* Artemisia capillaris* extract,* Schisandra chinensis* extract, and* Aloe vera* composition, N-931.* Artemisia capillaris* extract was produced as 70% ethanol extracts of the aerial parts with no less than 3% chlorogenic acid.* Schisandra chinensis* extract was standardized as 70% ethanol extract of the fruits containing at least 2% total schisandrins. N-931 is a unique combination of 1–4% aloesin and 96–99% 200 : 1* Aloe vera* inner leaf gel powder with not less than 8% polysaccharides provided by Aloecorp, Mexico.* Schisandra* extract,* Artemisia* extract, and* Aloe* N-931 were blended at a ratio of 4 : 8 : 3 to produce the standardized SAL composition containing no less than 0.2% schisandrins from* Schisandra chinensis* and 1.0% chlorogenic acid from* Artemisia capillaris* and N-931 with the above specifications.

### 2.2. Animals and Housing

Purpose bred female CD-1 mice, weighing 18–24 g, were purchased from USDA approved laboratory animal vendor (Charles River Laboratories, Inc., Wilmington, MA) and acclimated upon arrival for a week. Individual cages were identified with a cage card indicating project number, test article, dose level, group, and animal number. The Harlan T7087 soft cob bedding was used and changed at least twice/week. Animals were provided with fresh water and rodent chow diet #T2018 (Harlan Teklad, 370W, Kent, WA)* ad libitum* and were housed in a temperature controlled room (22.2°C) on a 12-hour light-dark cycle. All animal experiments were conducted according to institutional guidelines congruent with the guide for the care and use of laboratory animals and approved by IACUC with approval #SAL-441-14/15.

### 2.3. Model Inductions

A balanced therapeutic schedule was generated and optimized as follows to address prophylaxis and intervention: for APAP-induced hepatotoxicity model, APAP (Lot #MKBQ8028V, from Sigma) at a dose of 400 mg/kg dissolved in warm saline (Lot #132908 from G-Biosciences, Lot #720729 from Quality Biological) (heated to 60°C and cooled down to ambient temperature) was orally administered to overnight fasted CD-1 mice to induce toxicity [6, 7]. For the CCl_4_ induced hepatotoxicity model, CCl_4_ (Lot #SHBD5351V, from Sigma) at a dose of 25 *μ*L/kg dissolved in corn oil was administered intraperitoneally to overnight fasted CD-1 mice to induce toxicity [14, 22, 23]. For both models, materials were administered at −48 hr, −24 hr, and −2 hr before APAP or CCl_4_ administration and +6 hr after induction. Materials were administered at (a) 400 mg/kg, 325 mg/kg, and 250 mg/kg of composition SAL for dose-response study; (b) 106.7 mg/kg, 213.3 mg/kg, 80 mg/kg, and 400 mg/kg of* Schisandra chinensis*,* Artemisia capillaris*, N-931, and composition SAL, respectively, for synergy determinations; (c) 300 mg/kg of each* Schisandra chinensis*,* Artemisia capillaris*, N-931, and composition SAL for functional tests and comparative study; and (d) 400 mg/kg and 50 mg/kg of composition SAL and UDCA, respectively, for activity confirmation tests. In total, the mice received 3 doses before the chemical induction and a dose after the chemical induction. 10% Tween-20 (Lot #0134C141 from Amresco) was used as a carrier vehicle for all the materials. Control mice with or without APAP or CCl_4_ received carrier vehicle only.

### 2.4. Hepatic Function Test

Serum was isolated from blood drawn at T24 using serum separator tube after 30-minute room temperature clotting and spun at 3000 rpm for 10 minutes for ALT (alanine aminotransferase), AST (aspartate aminotransferase), total bilirubin, conjugated and unconjugated bilirubin, bile acid, total protein, albumin, globulin, and alkaline phosphatase monitoring in an automated colorimetric assay using Beckman Coulter AU2700 at Phoenix Laboratories (Everett, WA).

### 2.5. Glutathione (GSH) and Superoxide Dismutases (SODs) Measurements

Liver tissues were collected immediately after necropsy and were kept in dry ice until transferred to −80°C. Materials were then shipped to a contract laboratory (Brunswick Laboratories, 200 Turnpike Road, MA 01772, USA) in dry ice for final specimen processing and biomarker analysis. (A) Sample preparation: Frozen tissue was ground to a course powder using a pulverizer. 1 mL of PBS containing 19.6 *μ*M EDTA was added to ~0.2 g of ground tissue and homogenized for 1 min in ice bath using a homogenizer from Omni International. The mixture was then centrifuged for 15 min at 10,000 rpm at 4°C. A portion of the supernatant was used for SOD and protein analysis. The rest of supernatant was further processed as follows for GSH analysis. (B) GSH analysis: portion of the supernatant was mixed with the same volume of 100 mg/mL MPA solution to deproteinized in order to avoid interference from proteins. The mixture was allowed to stand at room temperature for 5 min after vortexing and then centrifuged for 15 min at 10,000 rpm at 4°C. The deproteinated supernatant was evaluated for GSH content. Glutathione (GSH) is a key intracellular tripeptide thiol that helps protecting cells from free radical damage by providing reducing equivalents for the reduction of lipid hydroperoxides. During this process, oxidized glutathione (GSSG) forms as a reaction product. GSH level has been used as indicative biomarkers of* in vivo* oxidant and oxidative stress level in cells and tissues. In this analysis, the sulfhydryl group of GSH reacts with DTNB (5,5′-dithio-bis-2-(nitrobenzoic acid)) to produce a a yellow colored 5-thio-2-nitrobenzoic acid (TNB) product. The amount of GSH in the deproteinated supernatant is determined via measurement of the absorbance of TNB at 410 nm. A Glutathione Assay Kit from CAYMAN Chemical Co., Inc. (Ann Arbor, Michigan), was used for analysis. (C) SOD and protein analysis: superoxide dismutases (SODs) are metalloenzymes that catalyze the dismutation of the superoxide anion to molecular oxygen and hydrogen peroxide. SOD is considered one of the most important antioxidant enzymes* in vivo*. The SOD assay is a colorimetric assay, which utilizes a tetrazolium salt to measure the dismutation of superoxide radicals that were induced by xanthine oxidase and xanthine, and the activity of SOD in a given sample is quantified by the standard curve generated using the SOD standards. One unit of SOD is defined as the amount of enzyme needed to exhibit 50% dismutation of superoxide radicals. A Superoxide Dismutase Assay Kit from CAYMAN Chemical Co., Inc., was used for analysis. The protein concentrations of the tissue homogenates were determined by assessing protein concentrations of the suppressants via a Pierce*™* BCA Protein Assay Kit. (D) Materials and equipment: Homogenizer (cat number TH-01) from Omni International (Kennesaw, GA); Hard Tissue Omni Tip*™* Plastic Homogenizing Probes (7 mm × 110 mm) from Omni International (Kennesaw, GA); Refrigerated Centrifuge (model number 5402) from Eppendorf (Hauppauge, NY); and Microplate Reader (model number Synergy HT) from US Biotek (Shoreline, WA) were used.

### 2.6. Histopathology

Liver samples were fixed in 10% buffered formaldehyde and embedded in paraffin wax for histological examination. All microsectioned (5 *μ*m) slides were stained with haematoxylin/eosin and analyzed under the microscope system (Olympus BX 51 microscope and DP72 digital camera, Olympus Optical Co., Ltd., Tokyo) with a magnification ×200.

### 2.7. Statistical Analysis

Data were analyzed using SigmaPlot (version 11.0, Systat Software, Inc., San Jose, CA). The results are represented as mean ± standard deviation. Statistical significance among groups was calculated by means of single factor analysis of variance (ANOVA) and by *t*-test. *p* values less than or equal to 0.05 (*p* ≤ 0.05) were considered as significant. When normality test failed, for nonparametric analysis, data were subjected to Mann-Whitney sum ranks for *t*-test and Kruskal-Wallis one-way ANOVA on ranks for ANOVA.

## 3. Results

### 3.1. Dose-Response Effect of SAL

The optimum dosage of the composition SAL that incurs significant liver protection was evaluated both in APAP and in CCl_4_ induced models. Mice were given orally the composition SAL at doses of 400 mg/kg, 325 mg/kg, and 250 mg/kg suspended in 10% Tween-20. As seen in Table 1, in the APAP group, dose-correlated inhibitions in serum ALT were observed for the composition. 52.5% (*p* = 0.004), 48.5% (*p* = 0.007), and 34.6% (*p* = 0.05) inhibitions were observed for mice treated with doses of 400 mg/kg, 325 mg/kg, and 250 mg/kg SAL, respectively. Similarly, in the CCl_4_ group, dose-correlated inhibitions in serum ALT were observed for the composition. 46.3% (*p* < 0.001), 39.5% (*p* = 0.003), and 29.9% (*p* = 0.02) inhibitions were observed for mice treated with doses of 400 mg/kg, 325 mg/kg, and 250 mg/kg SAL, respectively. There was a 100% survival rate for all the groups in both models. Compared to the normal control animals that received 10% Tween-20, administration of APAP and CCl_4_ caused a 229- and 496-fold increase in serum ALT, respectively. The composition SAL has provided statistically significant protection from liver damage at a dosage level as low as 250 mg/kg as determined by serum ALT level when compared to vehicle treated diseased mice.

### 3.2. Unexpected Synergy

The efficacy of individual plants was tested including* Schisandra*,* Artemisia*, and N-931 at a dosage equivalent to each plant ratio in the composition of SAL as they appear in 4S : 8A : 3L at the highest dose tested (400 mg/kg). An average of 20% inhibition with 70–80% survival rates was observed for these plants at the given dose. Colby's equation [24] was utilized to evaluate the benefit of combining* Schisandra chinensis*,* Artemisia capillaris*, and N-931 in both APAP and CCL_4_ model. As shown in Table 2, the observed values were greater than the expected hypothetical values in both the models indicating the existence of synergy in formulating three ingredients at a specific ratio in SAL. The merit of blending* Schisandra*,* Artemisia*, and N-931 was confirmed by their synergistic protection from liver damage caused by APAP and CCl_4_ induction.

### 3.3. Liver Protection Activity of Composition SAL Compared to Its Individual Components

**Figure 1:**
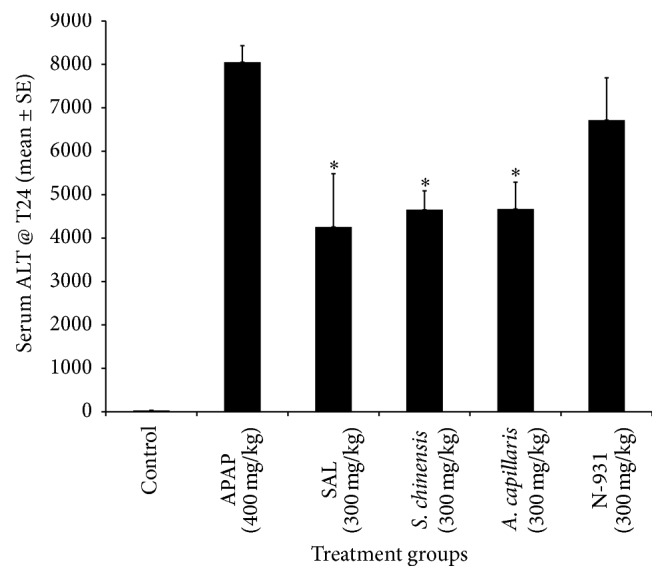
Liver protection activity of the composition of SAL against its individual components at a Dose of 300 mg/kg in APAP-induced hepatotoxicity model. ^*∗*^ *p* ≤ 0.05.

**Figure 2:**
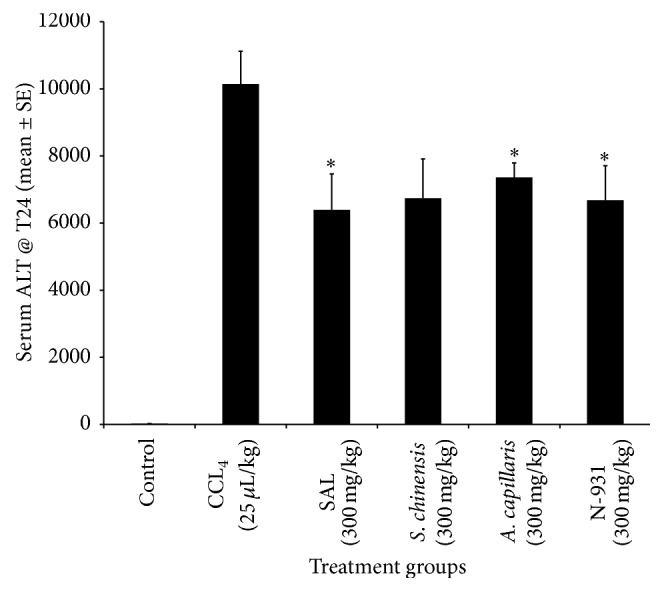
Liver protection activity of the composition of SAL against its individual components at a dose of 300 mg/kg in CCl_4_ induced hepatotoxicity model. ^*∗*^ *p* ≤ 0.05.

Both APAP and CCl_4_ induced liver toxicity models were utilized to compare the liver protection activity of the SAL composition against its individual components at a dose of 300 mg/kg using reduced serum ALT level as a measure of efficacy. As seen in Figures 1 and 2, the composition (SAL) showed enhanced liver damage protection compared to vehicle in the APAP model. Statistically significant 47.1, 42.2, 42.0, and 16.6% reductions in serum ALT were observed for mice treated with SAL,* Schisandra*,* Artemisia*, and N-931 compared to vehicle group, respectively. In this study, the lowest survival rate (50%) was observed for mice treated with* Artemisia* while survival rates of 90, 70, and 70 were observed for the composition,* Schisandra*, and N-931, respectively. Further substantiating the evidence observed in APAP model, the composition SAL showed greater liver protection than each individual component at a dose of 300 mg/kg in the CCl_4_ model using serum ALT as a measure of efficacy. Reductions of 37.0, 33.6, 37.4, and 34.2% were observed for SAL,* Schisandra*,* Artemisia*, and N-931, respectively. There was a 100% survival rate for all the groups in this model.

### 3.4. Moderation of Hepatic Functional Panel

Liver panels such as AST, ALT, total bilirubin, conjugated and unconjugated bilirubin, bile acid, total protein, albumin, globulin, and alkaline phosphatase have been used as a standard screen method for liver health. Both APAP and CCl_4_ induced liver toxicity models were utilized to compare the liver protection activity of the composition of SAL (400 mg/kg) against pharmaceutical drug ursodeoxycholic acid (50 mg/kg) using liver panel data as a measure of efficacy. In this report only the CCl_4_ data have been depicted for the UDCA comparison. As depicted in Tables 3 and 4, statistically significant moderations in these major biomarkers were observed when induced mice were treated with the composition of SAL at a dose of 400 mg/kg. In the APAP model, 60.6% and 80.2% reductions in serums AST and bile acid, respectively, were observed for mice treated with the composition when compared to vehicle treated induced mice. Statistically significant increases in serums albumin and total protein were observed in the same model. The 90% survival rate observed in the SAL treated group, compared to the 60% in the APAP model, is a reflection of liver protection that occurred as a result of the composition. In the CCl_4_ model, 34.0, 44.5, 26.6 and 63.6% reductions in serums ALT, AST, bile acid, and direct bilirubin, respectively, were observed for mice treated with the composition (Table 4). For the comparison purpose, the pharmaceutical drug UDCA (ursodeoxycholic acid) was tested and showed 25.0, 41.2, 4.0, and 45.5% reductions in serums ALT, AST, bile acid, and direct bilirubin, respectively, when compared to the vehicle control. Among these, only the AST and direct bilirubin values were statistically significant. These reductions were statistically significant. There was a 100% survival rate in the CCl_4_ models for both the intervention and vehicle treated groups.

### 3.5. Effect on Oxidative Stress Biomarkers in Liver Homogenates

Additional confirmatory assays were carried out to assess the effect of the composition of SAL in protecting liver using CCl_4_ induced hepatotoxicity model. Mice were given the composition SAL at 400 mg/kg. As shown in Table 5, the composition SAL replenished the depleted hepatic glutathione in association with an increase in hepatic superoxide dismutase. While an intraperitoneal injection of CCl_4_ at a dose of 25 *μ*L/kg to mice caused a 20.1% depletion in SOD, 51.3% increases in GSH were found in the liver tissues of these mice. The composition SAL restored the depleted SOD by 50.5% compared to vehicle treated CCl_4_ challenged mice. Similarly, a 25.9% increase in tissue GSH was also observed for these mice compared to vehicle treated CCl_4_ injected mice. The pharmaceutical drug, UDCA, increased the GSH and SOD level by 19.4% and 28.1% compared to the CCl_4_ administered animals. When these biomarker changes observed as a result of UDCA were subjected to a head-to-head comparison against the composition SAL, there were 5.56% and 17.5% increases in the GSH and SOD level, respectively, for the mice treated with the composition SAL, indicating the significance of the composition. These findings, in conjunction with previously disclosed liver panel data, strongly suggest that the SAL composition possesses liver protection activity from oxidative stress elicited by CCl_4_ induced liver damage.

### 3.6. Histopathology Findings

**Figure 3:**
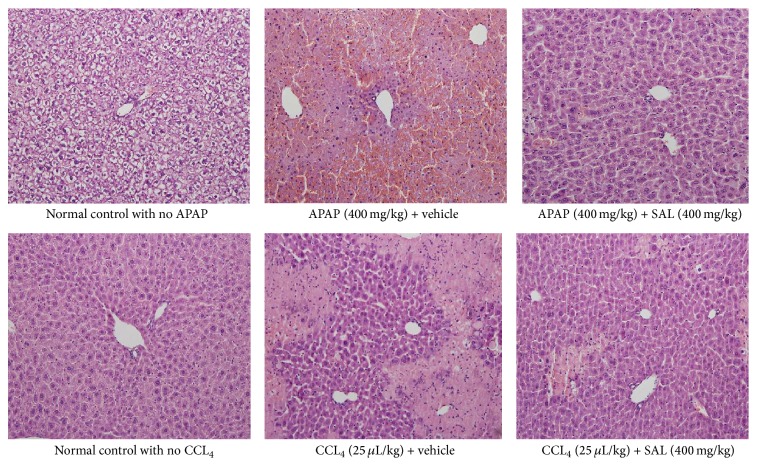
Histopathology of liver tissues from APAP and CCl_4_ induced hepatotoxicity models.

As seen in Figure 3, the liver tissues of the untreated control animals showed normal architecture of hepatic cells with clear cytoplasm, normal Kupffer cells, and normal large nuclei. In the vehicle treated APAP and CCl_4_ induced mice the liver tissue showed distorted architecture with extensive area of necrosis, cytoplasmic condensation, and marked nuclei shrinkage. Some degenerative ballooning and vacuolation were also observed in these groups. On the other hand, discernible normal cellular architecture and lesser degrees of structural changes were evident in mice treated with SAL in both models (Figure 3).

## 4. Discussions

Hepatoprotective plant extracts have been traditionally used for treatments of liver diseases over centuries. Furthermore, in modern pharmacotherapy, mounting experimental and clinical evidences suggest the hepatoprotective nature of Schisandrae. For example, oral pretreatment of rats with a lignan-enriched extract from the fruit of* Schisandra* at a dose of 1.6 g/kg for 3 days prevented carbon tetrachloride induced hepatotoxicity and glutathione depletion and stimulated the activity of glutathione reductase [12]. One of the active constituents of* Schisandra*, gomisin A, administered at an oral dose of 50 mg/kg an hour before acetaminophen injection resulted in statistically significant reduction in serums ALT and AST at 18 and 24 hours after induction minimized degeneration and necrosis also observed in this study [13]. In another publication, administration of gomisin A at a dose of 12.5–50 mg/kg in CCl_4_, d-galactosamine, and orotic acid induced hepatotoxicity also showed improved bile flow and liver function in rats [14]. Similarly pretreatment or concurrent administration of wuweizisu C to rats has showed reduced serum transaminase activities and improved histological changes such as fatty degeneration, cell necrosis, and inflammatory cell infiltration, in liver injuries induced by a single or repeated administration of carbon tetrachloride (CCl_4_), d-galactosamine, and dl-ethionine [25]. Schisandrol A or schisandrin B administered orally at a dose of 200 mg/kg for 3 days reduced liver malondialdehyde formation when mice were sacrificed 12 hours after liver toxicity induced by administration of 50% ethanol. These compounds also increased superoxide dismutase and catalase activities in rat liver cytosol and were able to inhibit gossypol-induced superoxide anion generation in rat liver microsomes [15]. The mechanism by which schisandrin B exerts its hepatoprotectant effect appears to be through the enhancement of the hepatic mitochondrial glutathione antioxidant status in mice with CCl_4_ induced hepatotoxicity [22]. Pretreating mice with schisandrin B at a daily dose of 1 mmol/kg for 3 days protected against menadione-induced hepatic oxidative damage in mice, as evidenced by decreases in plasma ALT (78%) and hepatic malondialdehyde level (70%), when compared with the menadione intoxicated control [26]. In CCl_4_ induced liver injury, the hepatoprotective effect of the major components isolated from Schisandrae is believed to be due to their inhibitory effect on lipid peroxidation and the binding of CC14-metabolites to lipids of liver microsomes [23].

More than 5000 case studies of hepatoprotective effects of fruit extracts from* Schisandra* have been reported in Chinese literature with patients with hepatitis; in a clinically controlled trial involving 189 patients with chronic viral hepatitis B and elevated ALT levels, an ethanol extract of the fruits, containing 20 mg of lignans (equivalent to 1.5 g of the fruits), resulted in 68% (72/107) and 44% (36/72) improvement in serum ALT levels within 4 weeks and 8 weeks, in patients receiving the extract and control group, respectively [16].

Besides the description of hepatoprotective effect from* Schisandra* extract, the physiological adaptogenic properties of extracts from* Schisandra* have also been reported. This mechanism will make the host prone to adapt to a state of nonspecific resistance which leads to biochemical changes at the time of exposure to harmful external or internal factors resulting in a more rapid and effective response to the stimuli [27]. By doing so, the extracts increase the host resistance to a wide range of physical, chemical, and emotional stresses while promoting improved overall moderation of physiological processes. Enhancement of liver protections observed in the compositions SAL could be, in part, contributed by the adaptogenic characteristics of* Schisandra*.

In addition to treating various hepatic disorders in traditional oriental medicine, significant experimental reports have been documented to show the hepatoprotective activities of* Artemisia capillaries* as the other active ingredient of the present composition. For example, when aqueous extract of* A. capillaries* was administered orally at a dose range of 50–100 mg/kg for 10 days to mice at which liver injuries were induced by oral administration of 30% alcohol (10 mL/kg, twice/day) plus pyrazole (PRZ, 30 mg/kg), statistically significant reductions in ALT, AST, and malondialdehyde (MDA) levels in serum and liver tissues were observed for mice treated with the extract. In addition, it (a) moderated microvesicular steatosis and necrosis in hepatic histopathology; (b) replenished the antioxidant components including glutathione content and total antioxidant capacity and activities of glutathione peroxidase (GSH-Px) and catalase and SOD; (c) normalized levels of tumor necrosis factor-alpha (TNF-*α*) and transforming growth factor-beta (TGF-*β*) in hepatic tissues; and (d) attenuated the alterations of aldehyde dehydrogenase (ALDH) level in serum and hepatic gene expressions of ALDH and alcohol dehydrogenase (ADH) were the principal markers positively impacted suggesting both enhancement of antioxidant activities and modulation of proinflammatory cytokines as the possible mechanisms that could be involved during the hepatoprotective activity of* A. capillaries* [17]. In 2,2-azobis(2-amidinopropane) dihydrochloride (AAPH) induced oxidative stress, rats pretreated with aqueous extract of* A. capillaries* for 7 days at a dose of 7.5 g/kg significantly attenuate serums ALT and AST and improved glutathione levels and enhanced the production of catalase and significantly attenuated the accumulation of thiobarbituric acid-reactive substances in both plasma and liver tissues compared with those of rats given AAPH alone [18]. In an optimal dosage finding study carried out using subacute hepatotoxicity model induced by 10-week injection of carbon tetrachloride to rats, aqueous extract of* A. capillaris* (at 200 mg/kg) resulted in moderations in transaminase activities, MDA, and hydroxyproline concentrations [28]. Knowing the fact that ethanol (EtOH) is almost exclusively metabolized by the liver, a human hepatoma cell lines (Hep G2 cell) were used to study effect of an aqueous extract of* A. capillaris* on alcohol-induced hepatotoxicity* in vitro*. In this study,* A. capillaris* at a concentration range of 0.5–5 *μ*g/mL inhibited the secretion of EtOH-induced interleukin-1*α* (IL-1*α*) and tumor necrosis factor-*α* (TNF-*α*), IL-1*α*, and TNF-*α*-induced cytotoxicity and inhibited the EtOH-induced apoptosis of Hep G2 cells [29]. In a bile duct ligation- (BDL-) induced cholestatic fibrosis model, aqueous extracts of* A. capillaris* administered daily at a dose of 25 or 50 mg/kg for two weeks significantly reduced serum malondialdehyde and liver hydroxyproline levels and restored depleted glutathione content and glutathione peroxidase activity and attenuate cholestatic liver injury and collagen deposition and suppressed expression of fibrogenic factors suggesting the antifibrotic properties through both upregulation of antioxidant activities and downregulation of extracellular matrix protein production [30].

The third ingredient used in formulation of the SAL composition was* Aloe vera* N-931, containing* Aloe* chromone aloesin and* Aloe* polysaccharide. Chromones isolated from various* Aloe* species have been reported to have diverse biological activity. Aloesin is a C-glycosylated 5-methylchromone with a potent antioxidation activity [21, 31]. In a recent study where the phytochemical profile of* Aloe barbadensis* was investigated using colorimetric assays, triple quadrupole and time-of-flight mass spectrometry, focusing on phenolic secondary metabolites in the different leaf portions, the outer green rind that contains aloesin was identified as the most active in radical scavenging activity, which is better than the inner parenchyma in stable radical DPPH test and ORAC assay. Further tests using isolated pure secondary metabolites confirmed that the 5-methylchromones aloesin were among the most active chromones [32].

Moreover, polysaccharides, the major constituents of* Aloe vera* gel, have been utilized for varieties of human disease and suggested for liver protection, in part, because of their antioxidant activities. For instance, strong antioxidant activities have been reported for purified polysaccharides from* Aloe barbadensis* gel when tested in DPPH, hydroxyl, and alkyl radical scavenging assays [33]. Similarly, in* Aloe* plant age and function related study, polysaccharides from three-year-old* Aloe* extract were found showing the strongest radical scavenging activity (72.19%) which was significantly higher than that of synthetic antioxidants butylated hydroxytoluene (70.52%) and *α*-tocopherol (65.20%) at the same concentrations of 100 mg/L via DPPH assay [34]. Polysaccharides isolated from* A. vera* have also been found to possess high antioxidant efficiency as demonstrated with a decrease in the oxidative stress marker MDA and an increase in the hepatic nonenzymatic antioxidant GSH and enzymatic antioxidant SOD* in vivo* in chronic alcohol-induced hepatotoxicity in mice [35].

Based on analyses of above results and data, it is reasonable to postulate that a composition comprised of these three plant materials possesses significant antioxidant activity and hence protects the liver from oxidative stress caused damage. To the best of our knowledge, these three plant extracts have never been reported to combine together before at specific ratios to formulate the SAL composition on the basis of literature search. In the present study, each component of the composition showed strong individual performances in modulating toxicities induced by the disclosed chemicals reinforcing the idea of combining these plant extracts for a better outcome in both models. This hypothesis needs to be confirmed in a way that the composition of SAL should demonstrate a boosted protection from liver damage elicited by both APAP and CCl_4_. When the combinations of these three plant materials were tested, clearly interesting yet, an unexpected synergy was observed from the SAL composition which exceeded the predicted effect based on simply summing the effect observed for each individual ingredient at the given ratio. In fact, none of the individual ingredients showed liver protection activity at the magnitude equivalent to the one noted for the composition in both models separately. Furthermore, data from liver function test including AST, ALT, bile acid, total protein, total bilirubin, conjugated bilirubin, albumin, and total protein demonstrated that the composition has indeed liver protection activity when compared to the vehicle treated control animals with liver injury. This is supported by the comparison of the effect of SAL composition against a pharmaceutical drug Ursodeoxycholic acid (UDCA). Moreover, as reflected from data of the liver homogenate, the composition SAL also replenished the depleted hepatic glutathione in association with an increased activity in hepatic superoxide dismutase. Glutathione is a key intracellular tripeptide thiol that helps protecting cells from free radical damage by providing reducing equivalents for the reduction of lipid hydroperoxides. Similarly, SODs are metalloenzymes that catalyze the dismutation of the superoxide anion to molecular oxygen and hydrogen peroxide. As a result, SOD is considered one of the most important antioxidant enzymes* in vivo*. These phase II enzymes substantiate each other to provide the strong antioxidant activity of the composition. These findings were substantiated by the histopathological observations signifying a liver protection capability of SAL composition in both APAP and CCl_4_ models.

## 5. Conclusions

Collectively, based on analyses of data from the hepatic function test, antioxidation biomarkers, and histopathological findings, we strongly believe that combining these traditionally well-known folk medicinal plants* Schisandra chinensis*,* Artemisia capillaris*, and* Aloe vera* N-931 into the ratio of 4S : 8A : 3L provided a significantly enhanced liver protection activity to the composition. Therefore, the composition of SAL could potentially be considered as a mitigating agent for alcohol and/or chemical induced hepatotoxicity.

## Figures and Tables

**Table 1:** Dose-correlated liver protection of the SAL composition in APAP/CCl_4_ induced hepatotoxicity model.

Composition	*N*	Dose (mg/kg)	Dose (mg/kg) L498/R684/N-931	APAP (400 mg/kg)		CCL_4_ (25 *µ*L/kg)	
				Serum ALT (mean ± SD)	*p *values	Serum ALT (mean ± SD)	*p *values
Control (−)	10	—	0	37.4 ± 8.7	—	21.4 ± 4.7	—

APAP/CCL_4_	10	—	0	8558.6 ± 2297.6	—	10616.4 ± 3386.3	—

SAL	10	250	66.7/133.3/50	5600.4 ± 3399.8	0.05	7445.3 ± 2472.2	0.02
	10	325	86.7/173.3/65	4406.0 ± 3040.5	0.007	6417.8 ± 2421.0	0.003
	10	400	106.7/213.3/80	4065.1 ± 2046.9	0.004	5697.3 ± 2697.4	<0.001

Mice (*n* = 10) were orally given composition SAL at doses of 250, 300, and 400 mg/kg at **−**48 hr, **−**24 hr, **−and **2 hr before APAP or CCl_4_ administration and +6 hr after induction of model suspended in 10% Tween-20. Hepatotoxicity models were induced using 400 mg/kg and 25 *µ*L/kg APAP and CCl_4_, respectively. APAP/CCL_4_: the vehicle group of mice was induced by APAP/CCL_4_ and did not receive the SAL. Serum ALT was determined at T24. Data are expressed as mean ± SD. L498 = *Schisandra*, R684 = *Artemisia*, N-931 = *Aloe* polysaccharide with aloesin.

**Table 2:** Unexpected synergistic effect of *Schisandra chinensis*, *Artemisia capillaris,* and N-931 in liver protection.

Composition	Material	Dose (mg/kg)	*N*	Percent inhibition of vehicle	
				APAP (400 mg/kg)	CCL_4_ (25 *µ*L/kg)
SAL	*Schisandra* (L498) (*X*)	106.7	10	18.4	17.5
	*Artemisia* (R684) (*Y*)	213.3	10	20.8	22.8
	*Aloe* (N-931) (*Z*)	80.0	10	20.8	15.0
	Expected^*∗∗*^	400	—	48.8	45.9
	Observed^*¥*^	400	10	52.8	46.3

Data of serum ALT are presented as percentage change of vehicle. Mice (*n* = 10) were given composition SAL (400 mg/kg), *Schisandra* (106.7 mg/kg), *Artemisia* (213.3 mg/kg), N-931 (80 mg/kg), and vehicle at −48 hr, −24 hr, and −2 hr and +6 hr after induction of model suspended in 10% Tween-20. ^*∗∗*^Calculated value according to Colby's equation = *A* + *B* − *C*; that is, *A* = (*X* + *Y* + *Z*), *B* = (*XYZ*)/10000, and *C* = ((*XY*)+(*XZ*)+(*YZ*))/100.  ^*¥*^Data observed when a composition was orally administered at 400 mg/kg, when observed ≥ expected = unexpected synergy.

**Table 3:** Effect of SAL (300 mg/kg) on the major biomarkers of liver in APAP model.

Group	Material dose (mg/kg)	*N*	Survival rate	Analyte				
				AST (U/L)	Bile acid (*μ*mol/L)	T. bilirubin (mg/dL)	Albumin (g/dL)	T. protein (g/dL)
Control	0	10	100	77.7 ± 28.3	1.0 + 0.0	0.1 + 0.0	2.67 + 0.09	4.70 + 0.24
APAP (400 mg/kg)	0	10	60	4707.7 ± 2899.1	76.2 + 24.8	0.5 + 0.2	2.33 + 0.20	4.43 + 0.22
SAL	300	10	90	1855.7 ± 1859.6^*∗*^	15.1 + 5.7^*∗*^	0.3 + 0.1^*∗*^	2.71 + 0.12^*∗*^	4.84 + 0.12^*∗*^

Mice (*n* = 10) were orally given composition SAL at doses of 300 mg/kg at −48 hr, −24 hr, and −2 hr before APAP administrations and +6 hr after induction of model suspended in 10% Tween-20. Hepatotoxicity models were induced using 400 mg/kg APAP administered orally. Serum was collected at T24. Data are expressed as mean ± SD. ^*∗*^ *p* ≤ 0.05.

**Table 4:** Effect of SAL (400 mg/kg) on the major biomarkers of liver in CCl_4_ model.

Analyte	Control (*n* = 10)	CCL_4_ (25 *µ*L/kg) (*n* = 9)	CCl_4_ (25 *µ*g/kg) + SAL (400 mg/kg) (*n* = 9)	CCl_4_ (25 *µ*g/kg) + UDCA (50 mg/kg) (*n* = 10)
ALT (U/L)	20.0 ± 6.5	9796.5 ± 2223.4	6466.6 ± 2696.5^*∗*^	7352.1 ± 3157.4
AST (U/L)	69.9 ± 16.1	5031.8 ± 1510.2	2794.0 ± 1427.2^*∗*^	2957.3 ± 1451.6^*∗*^
T. bilirubin (mg/dL)	0.17 ± 0.05	0.40 ± 0.11	0.31 ± 0.09	0.36 ± 0.10
Direct bilirubin (mg/dL)	0.00 ± 0.00	0.11 ± 0.03	0.04 ± 0.05^*∗*^	0.06 ± 0.05^*∗*^
Indirect bilirubin (mg/dL)	0.17 ± 0.05	0.29 ± 0.09	0.27 ± 0.07	0.30 ± 0.08
ALP (U/L)	76.6 ± 15.7	139.7 ± 65.5	115.0 ± 19.5	111.5 ± 33.7
Bile acid (*μ*mol/L)	1.2 ± 0.4	30.1 ± 8.6	22.1 ± 7.4^*∗*^	28.9 ± 12.2
T. protein (g/dL)	4.50 ± 0.19	4.62 ± 0.20	4.61 ± 0.18	4.63 ± 0.18
Albumin (g/dL)	2.42 ± 0.13	2.64 ± 0.07	2.60 ± 0.09	2.60 ± 0.12
Globulin (g/dL)	2.08 ± 0.14	1.98 ± 0.15	2.01 ± 0.18	2.03 ± 0.14

Mice (*n* = 10) were orally given composition SAL at doses of 400 mg/kg and UDCA at doses of 50 mg/kg at −48 hr, −24 hr, and −2 hr before intraperitoneal CCl_4_ injection and +6 hr after induction of model suspended in 10% Tween-20. Hepatotoxicity models were induced using 25 *µ*L/kg of CCl_4_. Serum was collected at T24. Data are expressed as mean ± SD. ^*∗*^ *p* ≤ 0.05.

**Table 5:** Effect of composition SAL on oxidative stress biomarkers in liver homogenates collected from CCl_4_induced hepatotoxicity model.

Group	Dose (mg/kg)	*N*	GSH (nmole/mg of protein)	SOD (U/mg of protein)
Control	0	10	38.26 ± 9.52	19.04 ± 4.20
CCl_4_ (25 *µ*L/kg)	0	9^a^	57.87 ± 10.85	15.21 ± 6.09
SAL	400	9^b^	72.91 ± 14.93^*∗*^	22.89 ± 7.95^*∗*^
UDCA	50	10	69.07 ± 10.09^*∗*^	19.48 ± 4.64

^*∗*^ *p* ≤ 0.05; ^a^Misdosed and hence data for one mouse was excluded; ^b^Not enough blood to match liver homogenate data and hence one mouse was excluded. UDCA = ursodeoxycholic acid. Mice received three doses of the composition before model induction and a single dose after model induction.
